# Odor Emissions from Raw Meat of Freshly Slaughtered Cattle during Inspection

**DOI:** 10.3390/foods10102411

**Published:** 2021-10-12

**Authors:** Francesca Conte, Fabrizio Cincotta, Concetta Condurso, Antonella Verzera, Antonio Panebianco

**Affiliations:** Department of Veterinary Sciences, University of Messina, Polo Universitario dell’Annunziata, Viale G. Palatucci, 98168 Messina, Italy; francesca.conte@unime.it (F.C.); fabrizio.cincotta@unime.it (F.C.); concetta.condurso@unime.it (C.C.); antonio.panebianco@unime.it (A.P.)

**Keywords:** freshly slaughtered cattle, raw meat, volatile compounds, HS-SPME-GC-MS

## Abstract

Raw meat of Freshly Slaughtered Cattle (FSC) is characterized by a very weak odor defined as slightly sweet, which could interfere in off-odor assessment during post-mortem inspection by an official veterinarian. Despite this, no information is reported in the literature on the volatiles which are emitted by FSC meat. Thus, this research aimed to study the volatile profile of raw cattle meat at different times within 24 h of slaughtering. Volatiles were analyzed and quantified using HS-SPME-GC-MS. γ-Butyrolactone, acetoin, and to a lesser extent, acid acetic were found to be the odor-active compounds of raw meat from freshly slaughtered cattle. Quantitative differences were observed up to 24 h; since the lowest levels of the odor-active compounds were reached between two and three hours from slaughtering, this period could be the most suitable for the post-mortem inspection process.

## 1. Introduction

Raw meat of Freshly Slaughtered Cattle (FSC) is characterized by a very weak odor and a mild serum-like taste [1]: it is described as salty, metallic, and bloody with a slightly sweet aroma. However, it constitutes a rich source of non-volatile precursors involved in the development of meat flavor. In Italy, the slightly sweet aroma of FSC is described as the odor of warm meat [2,3].

The odor of FSC is of great importance in the post-mortem inspection process, during which, the detection of possible odor anomalies can even lead to cattle carcass condemnations. Despite this, literature data only refer to cooked meat, and as regard the raw meat, do not consider the volatiles emitted within the first 24 h of slaughtering [4,5,6,7]. Off-odors may arise during the evisceration process or just after slaughter because of different factors, the most important of which are summarized in Table 1 and Table 2.

The main purpose of the inspection process is to ensure the safety and the quality of meat, including good meat sensory characteristics, namely color, odor, taste, and texture, as reported in the European legislation in force over the years. The Commission Implementing Regulation (EU) 2019/627 [16], in accordance with Regulation (EU) 2017/625 of the European Parliament and of the Council [17], as regards official controls, establishes that: “[…] carcasses and accompanying offal, shall be subjected to post-mortem inspection: (a) without delay after slaughter […]”. Otherwise, the European regulations in force provide, as reported above, that the carcasses must be subjected to post-mortem inspection without delay after slaughter and immediately refrigerated before distribution, except in those situations, justified by technological reasons, in which transport takes no more than 2 h [18,19].

The typical meat odor of FSC has always been regarded as a disturbing factor that negatively interferes with the off-odor assessment during carcass inspection.

Off-odors, even if rarely, could be the reason for the seizure and destruction of up to 2.2% of total slaughtered cattle condemnations [20,21].

Following up on the considerations reported above, some relevant questions arise:

- Which are the volatile compounds emitted by the meat of FSC that could interfere with the possible off-odor perception during the post-mortem inspection?
- How long does this odor last?

To the best of our knowledge, no information is reported in the literature on the volatiles emitted by the fresh meat immediately and in the first hours after slaughtering. In this context, the present research aims to identify the volatiles correlated with the slightly sweet odor of FSC meat.

## 2. Materials and Methods

### 2.1. Sampling

Twenty individual samples of the diaphragm (skirt) muscle from twenty cattle, half males and half females, were collected at a local municipal abattoir in Sicily immediately following animal evisceration in twenty days. The diaphragm was chosen because it is among the muscles with the most intense beef flavor [22]; furthermore, it can be taken quickly and easily.

The animals were randomly selected from crossbreed cattle, 18–24 months old, from farms very close to the abattoir. The animal feeding was constituted by polyphyte meadows plus a moderate integration of commercial feeds. All the animals were slaughtered immediately upon arrival and all were in a good health status, as was ascertained during the ante-mortem and post-mortem examination.

For the volatile extraction, a portion of about 300 g of skirt muscle, weighed with a precision of 0.01 g, was immediately transferred to a glass jar (77 mm diameter, 115 mm height), 1 mL of Internal Standard (IS) solution was added, and the jar was hermetically capped. The amount of meat was able to half-fill the jar. Each meat sample was placed into the jar, making sure to expose the serosa-free surface to the jar headspace.

Volatile compound extraction was performed immediately in the slaughterhouse and 1, 2, 3 and 5 h after slaughtering, maintaining the meat samples at room temperature. Subsequently, the samples were refrigerated (+4 °C) and analyzed 24 h after slaughtering. Each skirt muscle was analyzed in duplicate.

### 2.2. Volatiles Extraction

The headspace solid-phase microextraction (HS-SPME) technique was used for the aroma volatiles extraction. Extraction was performed in the headspace glass jar kept at room temperature (24 ± 1 °C) using a DVB/CAR/PDMS fiber of 50/30 μm film thickness (Supelco, Bellefonte, PA, USA), housed in its manual holder (Supelco, Bellefonte, PA, USA); this type of fiber is suitable for sampling all the volatile and semi-volatile compounds from 40 to 275 amu, even when present as trace.

The refrigerated samples were equilibrated at room temperature for 1 h prior the extraction. The fiber was then exposed to the sample headspace for 1 h. After the sampling, the SPME fiber was introduced onto the splitless injector of the GC/MS. The fiber was kept for 3 min in the injector port and maintained at 260 °C for the thermal desorption of the analytes onto the capillary GC column. No artifacts were observed after an SPME analysis of the headspace of an empty glass jar performed as a blank analysis.

For the optimization of the extraction technique, the effects of several variables, namely extraction time and desorption time and temperature, on the extraction efficiency were studied. The total peak area (total ion chromatogram) and the coefficient of variation (CV%) of the measurements were used to select the more appropriate extraction conditions in terms efficiency (data not reported).

### 2.3. Volatiles Analysis

For the analysis, a Shimadzu GC 2010 Plus gas chromatograph directly interfaced with a TQMS 8040 triple quadrupole mass spectrometer (Shimadzu, Milan, Italy) was used. The conditions were as follows: injector temperature, 260 °C; injection mode, splitless; capillary column, VF-WAXms, 60 m × 0.25 mm i.d. × 0.25 μm film thickness (Agilent, S.p.a. Milan, Italy); oven temperature, 45 °C held for 5 min, then increased to 80 °C at a rate of 10 °C/min and to 240 °C at 2 °C/min; carrier gas, helium at a constant flow of 1 mL/min; transfer line temperature, 250 °C; acquisition range, 30 to 360 m/z; scan speed, 1250 amu/s. The volatile compounds were identified using mass spectral data, NIST’ 18 (NIST/ EPA/NIH Mass Spectra Library, version 2.0, Gaithersburg, MD, USA) and the FFNSC 3.0 database, linear retention indices (LRI), literature data, and the injection of standards were available, as previously reported by Cincotta et al. [23].

### 2.4. Quantitative Analysis

Volatile compounds were quantified using the method of standard additions. In order to control for possible changes in the amount of the extracted analytes due to fiber adsorption–desorption, an aqueous solution of 1-hexanol was used as IS. Stock solutions of γ-butyrolactone, acetic acid and acetoin were prepared to dilute the corresponding standards (Merk, Merck Life Science S.r.l, Milan, Italy; purity ≥ 99%) in water (HPLC grade), whereas R-(+)-limonene (Merk, Merck Life Science S.r.l, Milan, Italy; purity ≥ 99%) and toluene (purity ≥ 99.9%; Merk, Merck Life Science S.r.l, Milan, Italy) were diluted in ethanol/water 1:1. Working solutions containing γ-butyrolactone, acetic acid, limonene, acetoin, and toluene in a ratio 0.6–1.4 times those of the corresponding analytes, plus a constant amount of IS (giving a final concentration in the meat sample of 1 ppm), were freshly prepared and added to multiple aliquots of the meat sample. The sample alone was also analyzed. For each analyte, quantitation was based on a five-point calibration curve generated by plotting the standard/IS detector response ratio versus the amount spiked of each standard. Each sample measurement was repeated two times. The samples were extracted and analyzed using HS-SPME–GC–MS, as described above.

### 2.5. Method Validation

The analytical method used for the meat volatile analysis was validated with respect to reproducibility, repeatability, accuracy, limit of detection (LOD), and limit of quantification (LOQ) using a synthetic meat matrix.

For repeatability (intraday precision), samples were analyzed five times consecutively, and for reproducibility (inter day precision), samples were analyzed twelve times in three nonconsecutive days; precision of the replicates was expressed as the relative standard deviation (RSD %). The accuracy of the method was evaluated through recovery studies that were performed using the matrix samples with 10 µg/g of acetoin and 1 µg/g of toluene, limonene, 2-methyl-3-buten-1-ol, p-cymene, 6-methyl-5-hepten-2-one, nonanal, acetic acid, γ-butyrolactone, and 1-nonanol. Three spiked samples were analyzed, and the results were expressed as percentage of recovery. The limits of quantification (LOQ) and detection (LOD) were calculated using specific calibration curves constructed using a synthetic meat matrix containing the analytes in the range of their concentration in the meat samples using the following formulas:

LOD = 3 × σ/m(1)

LOQ = 10 × σ/m(2)

where σ is the standard deviation of the intercept and m is the slope of the calibration curve.

### 2.6. Statistical Analysis

Data were statistically elaborated using the XLStat software, version 3, May 2014 (Addinsoft, Damremont, Paris, France). One-way ANOVA was performed to detect significant differences in the volatile amount and odor activity values (OAVs) at different times from slaughtering. The model was statistically significant with a *p*-value < 0.05.

## 3. Results and Discussions

The results of the method validation procedure (Table 3) proved that the developed method is effective for the determination and quantification of the aroma volatiles emitted by fresh-slaughtered raw meat.

The average content (µg/g of raw meat) of the volatile compounds released by meat at different times from slaughtering is reported in Table 4, along with their linear retention index (LRI) and odor threshold value (OTV).

The volatile profile of the raw meat of FSC was characterized by a small number of compounds; nine volatiles belonging to different classes of organic compounds were identified; among these, only toluene, limonene, acetoin (3-hydroxy-2-butanone), acetic acid, and γ-butyrolactone were quantifiable, whereas 2-methyl-3-buten-1-ol, 1-nonanol, p-cymene, and 6-methyl-5-hepten-2-one were present at trace levels.

Our results cannot be compared with the literature data since they mainly refer to cooked meat and those on raw meat do not consider the volatiles emitted in the first 24 h after slaughtering, as reported above. The limited number of volatiles identified in our samples is reasonable; in fact, it is known that raw meat, characterized by a weak odor, is just a reservoir of aroma and flavor precursor compounds and the intensive meat odor and aroma are thermally derived.

Regarding the identified volatiles, limonene originates directly from animal feeding, and since terpene biosynthesis occurs exclusively in the plant kingdom, its occurrence suggests the presence of green forage in the cattle diet [30,31]. Toluene also derives directly from animal feedstuffs, as previously reported by other authors [32]. γ-Butyrolactone is linked to the feeding system too, but in this case, there is no direct transfer from the ingested feeds into tissue; in fact, γ-butyrolactone is the final product of the oxidation of the dietary fatty acids that occurs in the rumen [30,31]. Higher lactone amounts were reported in the fat tissue of ruminants fed grain-based diets compared to grass-fed ruminants [33]. Finally, acetoin and acetic acid are associated with the activity of ruminal microorganisms since they are well-known products of rumen fermentation [34,35].

Quantitative differences were observed in the volatile profile of the meat samples analyzed at different times after slaughtering. Specifically, significant differences were found for the amount of γ-butyrolactone, acetoin and acetic acid: γ-butyrolactone decreased during all the considered periods, acetoin decreased up to 3 h and then increased, while acetic acid remained stable for up to 3 h and then increased.

OAVs were considered to evaluate the contribution of the identified compounds in developing the meat odor of FSC. For each volatile, the OAV was calculated as the ratio between the concentration of the volatile and its OTV: only the volatile compounds with a concentration equal or greater than their OTVs (thus, OAV ≥ 1) are capable of contributing to the raw meat aroma; the others must be considered odorless. Looking at Table 3, it is can be noted that the levels of toluene and limonene were always inferior to their OTVs, whereas the levels of acetoin and γ-butyrolactone were consistently higher than their OTVs; finally, acetic acid content exceeded its OTV only in the raw meat samples analyzed 5 and 24 h after slaughtering.

**Figure 1:**
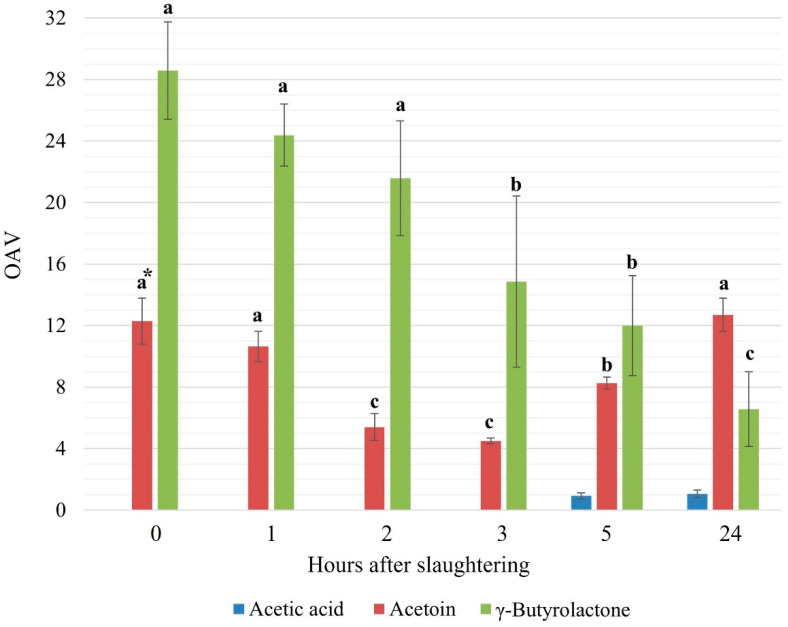
Average OAV for the main volatiles in meat samples at different times (hours) after slaughtering. * Different letters on bars of the same colors represent significant differences at *p* < 0.05 by Duncan’s multiple range test.

So, the only aroma active compounds in the raw meat samples of FSC were acetoin, γ-butyrolactone, and, limited to the meat samples analyzed at 5 h and after refrigeration up to 24 h after slaughtering, acetic acid. The aroma of raw cattle meat (Figure 1) up to 3 h after slaughtering is exclusively due to γ-butyrolactone and acetoin, with γ-butyrolactone as the major contributor.

The odor of γ-butyrolactone is described as sweet, pleasant, and creamy; similarly, acetoin is characterized by buttery, creamy, fatty, and sweet odor notes. After 5 and 24 h from slaughtering, acetic acid also contributed with its pungent notes to the odor of raw meat. However, due to its low OAV, acid acetic contributed to a lesser extent, whereas acetoin and γ-butyrolactone remained the compounds mainly responsible for the odor of these samples. So, according to these results, the volatiles emitted by the raw meat are present in the lowest amount between 2 and 3 h after slaughtering.

## 4. Conclusions

Following the results reported above, it is possible to affirm the FSC raw meat odor is due to a limited number of volatiles, mainly γ-butyrolactone and acetoin, which are characterized by sweet, pleasant, creamy, and buttery notes. The olfactive notes of these two volatiles are compatible with the definition of the FSC raw meat odor as slightly sweet. Furthermore, these aroma-active compounds showed the lowest values between two and three hours after slaughtering. Considering this, it is possible to conclude that this period could be the best window of time in which an official veterinarian can carry out a carcass off-odor assessment with the lowest interference of the basic odor of meat when a doubtful valuation arises immediately after slaughtering.

## Figures and Tables

**Table 1:** Carcasses’ off-odors deriving from various conditions.

Off-Odor	Origin	References
Manure	Late-gutted animals	[8]
Repugnant	Ingestion of wild garlic, absinthe, fish, rotten meat, rancid oil cake, Greek hay	[8]
Renetta apple	Tired animals after transport	[8]
Worms	Helminth infections in animals	[8]
Fishy	Animals fed ratios high in fish products	[9]
Anomalous	Ingestion of ether, chloroform, turpentine essence, chlorine, creolin, camphor, iodine, anise, kummel oil, sulfur, carbolic acid, linseed oil Some substances may be inhaled when stables or trucks are sanitized	[10]
Rancid	Protracted feeding of linseed, rapeseed, turnip-seed Fermented or spoiled pumpkins	[10]
“Sui generis”	Unripe carobs or residues from industrial processing of carobs Industrial citrus fruit by-products Garlics, onions, crocuses, forage kale	[10]
Urinous	Inadequate supply of drinking water	[10]
Unpleasant, disgusting fetid	Chrysalis infection	[10]
Amniotic fluid	Pregnant cows or that have recently given birth	[11]
Cat-like	Anomalous smell acquired during carcass refrigeration Presence of dirt in varnishes, paints, or packaging materials	[11]
Urinous	Exhausted animals	[12]
Repellent, pig manure-like	Greek hay (Trigonella foenum graecum) ingestion	[12]
Anomalous	Inhaled creolin or paint	[12]
Anomalous	Ingestion of turnip, silage from poultry litter, spoiled orange, and onion peels	[13]
Phosphorus-like	Ingestion of fresh onion leaves	[13]
Unpleasant	Ingestion of dieldrin-treated feed	[13]
Fecaloid (abdominal muscles and diaphragm)	Delayed evisceration Inadequate bleeding	[10]
Something sweet and unpleasant	Cows around the time of parturition	[13]

**Table 2:** Carcasses’ off-odors deriving from pathologies.

Off-Odor	Origin	References
Urinous, Ammonia	Uremia	[10]
Urinous	Urolithiasis	[10]
Fecaloid	Hepatogenic jaundice	[10]
Fecaloid	Hepatic pathologies	[10]
Putrid	Traumatic pericarditis, with large purulent or ichorous–purulent areas	[10]
Ammonia	Traumatic pericarditis	[10]
Putrid or putrid and ammonia	Ichorous–purulent peritonitis Sero-fibrinous peritonitis Multiple abscesses within the peritoneal cavity Gangrenous metritis, mastitis, and pneumonia	[10]
Peptic	Acute tympany	[10]
Fecaloid	Hepatic diseases	[10]
Putrid near the utero or in the abdominal regions	Septic metritis and metroperitonitis	[10]
Persistent of acetone, coinciding with birth	Ketosis in caws	[10]
Repellent and similar to a mixture of ether, ammonia, and methyl alcohol Butyric acid	Calf ascariasis	[10,11,12,13,14]
Rancid butter	Blackleg Malignant edema	[10]
Anomalous	Bovine slaughtered after a long transport	[10]
Cheese-like	Clostridial diseases	[10]
Something sweet	Ketosis in caws	[11]
Fluid with a rancid smell	Ketosis in caws	[10]
Sharp metallic odor (active lesion)	Parafilaria bovicola infection	[15]
Anomalous	Jaundice, kidney diseases, placental retention	[12]
Something sweet and unpleasant	Fluid build-up in meat, carcasses of feverish animals Ketosis or acetonemia	[13]
Unpleasant	Gangrenous injury	[13]

**Table 3:** Quality parameter of volatile compounds detected under the optimized HS-SPME-GC–MS method.

Compounds	Precision		Recovery (%)	LOD (µg/g)	LOQ (µg/g)
	Intraday RSD%	Interday RSD%			
Toluene	2.26	3.12	99.6	0.025	0.081
Limonene	3.41	4.86	99.7	0.003	0.010
2-Methyl-3-buten-1-ol	4.56	2.95	99.0	0.019	0.063
p-Cymene	2.27	4.26	99.3	0.014	0.046
Acetoin	4.96	3.71	99.1	0.038	0.123
6-Methyl-5-hepten-2-one	5.48	5.19	99.8	0.022	0.071
Nonanal	3.42	2.73	99.4	0.017	0.05
Acetic acid	2.38	4.25	99.3	0.028	0.094
γ-Butyrolactone	5.76	3.32	99.5	0.015	0.048
1-Nonanol	2.94	3.69	99.7	0.011	0.036

**Table 4:** Volatile compounds (µg/g of raw meat) released by meat at different times from slaughtering.

Compounds	LRI ^1^	Time from Slaughtering/Hours												Odor	OTV ^4^	
		0 ^2^		1 ^2^		2 ^2^		3 ^2^		5 ^2^		24 ^3^				
		X ^5^	SD	X	SD	X	SD	X	SD	X	SD	X	SD		ppm	Ref.
Toluene	1046	0.53	0.32	0.55	0.39	0.45	0.43	0.23	0.01	0.39	0.11	0.49	0.18	Sweet, pungent, benzene-like	1.0–2.9	[24]
Limonene	1150	0.19a ^6^	0.08	0.26a	0.01	0.28a	0.05	0.30a	0.03	0.11b	0.03	tr c	-	Citrus	1.2	[25]
2-Methyl-3-buten-1-ol	1245	tr ^7^	-	tr	-	tr	-	tr	-	tr	-	tr	-	Butter, sweet, balsamic	0.25	[24]
p-Cymene	1287	tr	-	tr	-	tr	-	tr	-	tr	-	tr	-	Citrus, fresh, solvent	0.12	[25]
Acetoin	1294	9.83a	2.40	8.52a	1.28	4.32c	1.39	3.61c	0.30	6.61b	0.30	10.15a	1.72	Buttery, creamy, dairy, milky, fatty, sweet	0.8	[24]
6-Methyl-5-hepten-2-one	1350	tr	-	tr	-	tr	-	tr	-	tr	-	tr	-	Citrus, lemon	1	[26]
Nonanal	1396	tr b	-	tr b	-	tr b	-	tr b	-	tr b	-	0.75 a	0.20	Waxy, aldehydic, rose, fresh, orris, orange peel, fatty, peel, green, cucumber	1	[24]
Acetic acid	1460	0.73b	0.17	0.98b	0.02	0.98b	0.19	0.81b	0.19	1.81a	0.19	2.05a	0.93	Pungent, acidic, cheesy, vinegar	1.958	[27]
γ-Butyrolactone	1640	1.00a	0.22	0.85	0.26	0.76a	0.14	0.52ab	0.19	0.42b	0.17	0.23b	0.17	Sweet, toast, caramel	0.035	[28]
1-Nonanol	1666	tr	-	tr	-	tr	-	tr	-	tr	-	tr	-	Rose-orange	1	[29]

^1^ Linear retention indices calculated on VF-WAXms column according to Van den Dool and Kratz equation. ^2^ Meat samples maintained at room temperature. ^3^ Meat samples maintained at + 4 °C starting from 3 h after slaughtering. ^4^ Odor threshold value. ^5^ Average value of ten meat samples, each in duplicate. ^6^ Different letters in the same row represent significant differences at *p* < 0.05 by Duncan’s multiple range test. ^7^ < to 0.01 ppm.

## Data Availability

Not applicable.
